# Influenza Immunization at Midlife and the Risk of Parkinson Disease

**DOI:** 10.1001/jamanetworkopen.2025.47140

**Published:** 2025-12-05

**Authors:** Antonios Douros, Ying Cui, Sophie Dell’Aniello, Samy Suissa, Paul Brassard

**Affiliations:** 1 Institute of Clinical Pharmacology and Toxicology, Charité–Universitätsmedizin Berlin, Berlin, Germany; 2 Department of Epidemiology, Biostatistics, and Occupational Health, McGill University, Montreal, Quebec, Canada; 3 Centre for Clinical Epidemiology, Lady Davis Institute, Montréal, Quebec, Canada; 4 Department of Medicine, McGill University, Montréal, Quebec, Canada

## Abstract

**Importance:**

Influenza infection could be associated with a long-term increase in the risk of Parkinson disease (PD). However, the benefit of influenza immunization as a preventive measure for PD remains unknown.

**Objective:**

To assess whether immunization for influenza at midlife (between age 40 and 50 years) is associated with a decreased risk of PD.

**Design, Setting, and Participants:**

This cohort study used electronic medical records from the UK’s Clinical Practice Research Datalink Aurum. The study cohort comprised individuals vaccinated for influenza between 40 and 50 years of age (hereafter, with influenza immunization at midlife) from 1995 to 2017 and unvaccinated controls (hereafter, without influenza immunization at midlife) matched 1:1 on age, sex, socioeconomic status, and calendar month of the vaccination. All analyses were conducted between January and May 2025.

**Main Outcomes and Measures:**

The primary outcome was incident PD. A modified intention-to-treat exposure definition with a 2-year lag period was applied to assess the risk of incident PD associated with influenza immunization at midlife vs no influenza immunization at midlife. Cox proportional hazards regression models estimated hazard ratios (HRs) and 95% CIs of the study outcome. Inverse probability of censoring weighting was used to account for selection bias, and propensity score matching was used for confounding control. Secondary analyses assessed potential effect size modifiers. Sensitivity analyses explored the implications of different potential biases.

**Results:**

The study cohort included 1 191 209 individuals (mean [SD] age, 44 [3] years; 673 920 females [56.6%]), of whom 612 974 received influenza immunization at midlife and 578 235 did not receive influenza immunization at midlife . Influenza immunization at midlife vs lack thereof was not associated with the risk of PD overall (crude incidence rates per 1000 person-years, 0.16 vs 0.10; matched HR, 0.96; 95% CI, 0.76-1.22). Results varied over time, with the lowest point estimate approximately 8 years after vaccination (HR, 0.75; 95% CI, 0.52-1.08), but none of the differences were statistically significant. Results also varied by seasonality, with a lower point estimate for those vaccinated during influenza season (matched HR, 0.62; 95% CI, 0.33-1.15) compared with those vaccinated outside of influenza season (matched HR, 1.07; 95% CI, 0.81-1.42). Stratification by age, sex, or vaccination prior to cohort entry did not modify the association. Sensitivity analyses supported the findings of the primary analysis.

**Conclusions and Relevance:**

This cohort study found that influenza immunization at midlife was not associated with the risk of PD in the overall population. Potential benefits for PD risk occurring several years after vaccination or in specific patient subgroups require further investigation.

## Key Points

**Question:**

Is immunization for influenza between age 40 and 50 years associated with a decreased risk of Parkinson disease later during the life course?

**Findings:**

In this cohort study of more than 1 million individuals who received influenza immunization at midlife and their matched controls, influenza immunization at midlife was not associated with the risk of incident Parkinson disease overall. Although results varied based on seasonality and time since vaccination, none of the differences were statistically significant.

**Meaning:**

Findings of this study suggest that midlife influenza vaccination is not associated with the risk of Parkinson disease in the overall population, but potential benefits occurring several years after vaccination or in specific subgroups require further investigation.

## Teaser

This cohort study examines the risk of Parkinson disease among individuals who received and did not receive influenza vaccination between the ages of 40 and 50 years.

## Introduction

Parkinson disease (PD) is the second most common neurodegenerative disease worldwide, affecting more than 1 million people in the US alone.^1^ The incidence of PD increases with advancing age and is higher among men than women.^2^ The pathology of PD is related to loss of nigrostriatal dopaminergic neurons and the formation of Lewy bodies, which are α-synuclein–containing proteinaceous aggregates in neurons of the substantia nigra.^3^ However, the etiology of PD remains largely unclear.^3^ As a result, there are no curative or disease-modifying treatments available, and approved medications mainly alleviate symptoms, such as hypokinesia, rigor, or tremor.^4^

A potential etiologic mechanism for PD is based on infections, with different bacteria, viruses, and fungi having been suggested as possible causative pathogens over the years.^5^ The influenza virus is one of the most prominent pathogens in this regard, with preclinical studies suggesting its involvement in the degeneration of dopaminergic neurons and the promotion of α-synuclein phosphorylation and aggregation.^6,7^ Moreover, several observational studies have shown that influenza infection was associated with an increased risk of PD.^8,9,10^ A case-control study with a long follow-up also found that influenza infection was associated with an up to 73% increased risk of PD 10 years after infection,^10^ which is in line with current evidence regarding a long-term pathogenic process of PD.^3,11^

Given the accumulating evidence, there is a need to understand the potential role of immunization for influenza at midlife as a preventive measure against the development of PD later during the life course. The only population-based study in the area was limited by its small sample size and insufficient follow-up, which led to inconclusive findings and further precluded the assessment of the outcomes of immunization at midlife.^8^ To address this important knowledge gap, we conducted a large, population-based cohort study with almost 30 years of follow-up to assess whether immunization for influenza at midlife (between ages 40 and 50 years) is associated with a decreased risk of PD.

## Methods

### Data Source

The data source for this population-based cohort study was the UK’s Clinical Practice Research Datalink (CPRD) Aurum. The CPRD contains primary care data of more than 40 million patients, including 14 million who are currently registered; these patients are seen across 1370 general practices that account for 15% of such practices in the UK.^12^ In the UK, general practitioners receive reports from specialists and other clinicians and have a gatekeeping function in the health care system. The CPRD is a database with clinically rich data containing all drug prescriptions issued by general practitioners, vaccinations, medical diagnoses, laboratory test results, anthropometric variables (eg, body mass index [BMI]), and lifestyle variables (eg, smoking habits and alcohol consumption). For this study, the CPRD was linked to the practice level Index of Multiple Deprivation, an area-level measure of deprivation including income, employment, educational level, health, crime, barriers to housing and services, and living environment.^13^ The Independent Scientific Advisory Committee of the CPRD and the Jewish General Hospital Research Ethics Board (Montréal, Quebec, Canada) approved the study protocol and waived the informed consent requirement because the data were deidentified. We followed the Strengthening the Reporting of Observational Studies in Epidemiology (STROBE) reporting guideline.

### Study Cohort

The study cohort was formed by initially identifying individuals who were enrolled in the CPRD and received an influenza vaccine between 40 and 50 years of age (hereafter, with influenza immunization at midlife) from January 1, 1995, to December 31, 2017. They were matched 1:1 with individuals without influenza immunization at midlife who were selected from all enrolled in the CPRD on the basis of calendar month of the vaccination, age, sex, and CPRD general practice (to account for area-level socioeconomic status via Index of Multiple Deprivation quintiles). All members in the study cohort were required to have at least 1 year of medical history in the CPRD prior to cohort entry (to measure potential confounders) and at least 5 years of follow-up (to reduce the initial size of the cohort and allow sufficient observation time for the potential longer-term outcome of the vaccine). We excluded individuals with prior PD diagnosis or pharmacological treatment (levodopa, oral dopamine agonists, monoamine oxidase type B inhibitors, catechol-O-methyltransferase inhibitors, and apomorphine). The date of cohort entry for individuals with influenza immunization at midlife was defined as the date of first vaccination for influenza between 40 and 50 years of age. The date of study cohort entry for individuals without influenza immunization at midlife was within the same calendar month as for the other group, given that calendar month was one of the matching criteria. Individuals without influenza immunization at midlife were allowed to receive immunization for influenza at a later time.

### Exposure and Outcome 

We used a modified version of the intention-to-treat approach that was specifically adapted to the study question. The intention-to-treat approach was chosen given previous evidence suggesting that a single influenza infection may increase the risk of PD.^10^ Moreover, we imposed a 2-year lag for the study cohort to account for diagnostic delays related to PD and for biological plausibility.^3,11^ Hence, PD events occurring during the first 2 years of follow-up were discarded. The cohort was followed up from the end of the lag period until the occurrence of the outcome, exposure to influenza vaccine (only for individuals without influenza immunization at midlife), the end of registration with the general practice in the CPRD, death, or the end of the study period (December 31, 2023), whichever occurred first.

The outcome was incident PD, defined using diagnostic codes in the CPRD. These diagnostic codes have previously been validated showing high positive predictive values (>80%).^14^

### Covariates and Propensity Scores

In addition to the aforementioned matching variables, we considered BMI (calculated as weight in kilograms divided by height in meters squared; <25, 25-29, ≥30, or unknown) and smoking status (current, former, never, or unknown), using the last measurement before cohort entry. We also considered several comorbidities measured ever before cohort entry, including alcohol-related disorders, arterial hypertension, congestive heart failure, coronary artery disease, ischemic stroke or transient ischemic attack, hyperlipidemia, diabetes, chronic kidney disease, traumatic brain injury, cancer, and dementia. Additionally, we considered use of medications in the last year before cohort entry, including antidepressants, oral anticoagulants, antiplatelet agents, nonsteroidal anti-inflammatory drugs, and antipsychotics.

Using multivariable logistic regression, we calculated the propensity score (PS) for each person in the cohort based on the covariates. PS defined the probability of receiving immunization for influenza at midlife vs not receiving immunization for influenza at midlife. PS matching was conducted in a 1:1 fashion using a caliper width equal to 0.1 of the SD of the logit of the PS.^15^

### Statistical Analysis

We calculated the incidence rates (IRs) and 95% CIs of incident PD diagnosis for each influenza vaccine exposure category before and after PS matching using Poisson regression. In the matched cohort, we generated cumulative incidence curves of incident PD for each exposure category. Cox proportional hazards regression models estimated PS-matched hazard ratios (HRs) and 95% CIs of incident PD associated with immunization for influenza at midlife vs no immunization for influenza at midlife. When designing the study, we hypothesized that a relatively high number of individuals from the comparator group would get censored due to receiving immunization for influenza during follow-up. Because high rates of censoring in the comparator group only could introduce selection bias, we used inverse probability of censoring weighting (IPCW),^16^ an approach that was applied in studies on COVID-19 vaccines and on widespread vaccination programs.^17^ IPCW included all the aforementioned baseline covariates and potential factors of immunization-related censoring (eg, asthma and chronic obstructive pulmonary disease) as time-dependent covariates updated every 180 days. We used 2 Cox proportional hazards regression models for weight estimation: one model including only baseline covariates, and the other model including both baseline and time-dependent covariates. The ratio of the probabilities estimated by the 2 models was used to stabilize the weights in the outcome model. Stabilized weights were further truncated at the first and 99th percentiles. Weights were set equal to 1 in individuals with influenza immunization at midlife, while weights were allowed to vary in individuals without influenza immunization at midlife.

We conducted 4 secondary analyses to explore potential effect size modifiers. First, we stratified by age (40-44 years and 45-50 years) and sex. Second, we assessed the implications of seasonality, stratifying based on whether immunization at midlife occurred during influenza season or not. For this analysis, we used UK-specific data for the timing of influenza seasons.^18^ Third, we assessed the implications of very early immunization, stratifying based on whether individuals had received an influenza vaccine before cohort entry. Fourth, we assessed a potential time-response association, with time since immunization at midlife as the underlying time axis and thereby modeling time since immunization at midlife flexibly using cubic splines with 3 interior knots.

We conducted several sensitivity analyses to assess the robustness of our findings. First, we used alternate lag periods of 3, 5, and 10 years given the uncertainty about the latency of a potential association between immunization for influenza at midlife and the development of PD. Second, we used a stricter outcome definition by requiring at least 2 prescription medications for PD (levodopa, oral dopamine agonists, monoamine oxidase type B inhibitors, catechol-O-methyltransferase inhibitors, and apomorphine) within 6 months of the PD diagnosis. In this analysis, the date of the outcome was defined as the latest date between the date of PD diagnosis and the second PD drug prescription. Third, we repeated the analyses without applying IPCW. Fourth, we excluded individuals without immunization for influenza at midlife but who had received an influenza vaccine prior to cohort entry along with their matched pairs. All analyses were conducted between January and May 2025 using SAS 9.4 (SAS Institute) with statistical significance set at α = .05.

We conducted 2 post hoc analyses to assess potential dose-dependent associations. Using the cohort prior to PS matching, we created 4 exposure groups: 0 vaccinations (reference group), 1 vaccination, 2 to 7 vaccinations, and 8 or more vaccinations. The cutoffs for the exposure groups were based on the distribution of vaccinations in the cohort. Applying a time-varying exposure definition, we allowed individuals to contribute person-time to more than 1 exposure group during follow-up. To account for the insidious nature of the outcome, we retained the 2-year lag at cohort entry and used additional 2-year lags every time individuals changed their exposure group. In the first analysis, we adjusted only for baseline confounders using a time-dependent Cox proportional hazards regression model. In the second analysis, we used a marginal structural Cox proportional hazards regression model with all baseline covariates as well as BMI and comedications as time-dependent covariates updated every year.

## Results

Our study cohort included 1 191 209 individuals, of whom 612 974 received an influenza immunization at midlife and 578 235 did not receive an influenza immunization at midlife (eFigure 1 in Supplement 1). Before PS matching, the cohort had a mean (SD) age of 44 (3) years and consisted of 673 920 females (56.6%) and 517 289 males (43.4%). Compared with the comparator group, individuals with influenza immunization at midlife were more likely to be obese; be diagnosed with arterial hypertension, coronary artery disease, hyperlipidemia, diabetes, or chronic kidney disease; and have used antidepressants or antiplatelet agents in the past year (Table 1). After PS matching, covariates were well-balanced between the 2 groups, with all standardized mean differences being lower than 10%.

**Table 1.** Baseline Characteristics of Cohort Members Before and After Propensity Score Matching

Characteristic	Before PS matching, No. %		SMD	After PS matching, No. (%)		SMD
	With influenza immunization at midlife (n = 612 974)	Without influenza immunization at midlife (n = 578 235)		With influenza immunization at midlife (n = 87 562)	Without influenza immunization at midlife (n = 87 562)	
Age, mean (SD), y^a^	44.18 (3.46)	44.19 (3.46)	NA	43.85 (3.37)	43.85 (3.37)	NA
Sex						
Female^a^	348 608 (56.9)	325 312 (56.3)	NA	53 263 (60.8)	53 262 (60.8)	NA
Male	264 366 (43.1)	252 923 (43.7)	NA	34 299 (39.2)	34 300 (39.2)	NA
Index of multiple deprivation^a^						
1	97 352 (15.9)	92 758 (16.0)	NA	17 163 (19.6)	17 163 (19.6)	NA
2	96 645 (15.8)	91 677 (15.9)	NA	15 493 (17.7)	15 493 (17.7)	NA
3	127 574 (20.8)	120 581 (20.9)	NA	18 595 (21.2)	18 595 (21.2)	NA
4	134 219 (21.9)	126 654 (21.9)	NA	17 675 (20.2)	17 675 (20.2)	NA
5	157 184 (25.6)	146 565 (25.4)	NA	18 636 (21.3)	18 636 (21.3)	NA
BMI						
<25	178 265 (29.1)	196 313 (34.0)	−0.105	37 208 (42.5)	37 842 (43.2)	−0.015
25-29	166 920 (27.2)	145 156 (25.1)	0.048	24 702 (28.2)	23 843 (27.2)	0.022
≥30	171 094 (27.9)	88 357 (15.3)	0.311	7570 (8.7)	7798 (8.9)	−0.009
Unknown	96 695 (15.8)	148 409 (25. 7)	−0.246	18 082 (20.7)	18 079 (20.7)	<0.001
Smoking status						
Current	135 212 (22.1)	122 739 (21.2)	0.020	15 718 (18.0)	15 940 (18.2)	−0.006
Former	112 596 (18.4)	85 012 (14.7)	0.099	12 554 (14.3)	12 583 (14.4)	−0.001
Never	311 767 (50.9)	282 785 (48.9)	0.039	47 888 (54.7)	47 700 (54.5)	0.004
Unknown	53 399 (8.7)	87 699 (15.2)	−0.200	11 402 (13.0)	11 339 (13.0)	0.002
Comorbidities						
Alcohol-related disorders	84 341 (13.8)	59 678 (10.3)	0.106	7913 (9.0)	7384 (8.4)	0.022
Arterial hypertension	95 635 (15.6)	36 377 (6.3)	0.302	2629 (3.0)	2599 (3.0)	0.002
CHF	7981 (1.3)	868 (0.2)	0.136	23 (0.0)	18 (0.0)	0.006
CAD	27 074 (4.4)	2721 (0.5)	0.258	74 (0.1)	89 (0.1)	−0.007
Ischemic stroke or TIA	10 126 (1.7)	2013 (0.4)	0.131	155 (0.2)	110 (0.1)	0.013
Hyperlipidemia	102 083 (16.7)	25 469 (4.4)	0.407	1607 (1.8)	1672 (1.9)	−0.005
Diabetes	105 057 (17.1)	11 981 (2.1)	0.529	418 (0.5)	405 (0.5)	0.003
CKD	31 226 (5.1)	5799 (1.0)	0.240	234 (0.3)	189 (0.2)	0.010
TBI^b^	324 (0.1)	81 (0.0)	0.023	35 (0.0)	6 (0.0)	0.019
Cancer	19 187 (3.1)	7462 (1.3)	0.125	323 (0.4)	309 (0.4)	0.003
Dementia^b^	89 (0.0)	0 (0.0)	0.014	10 (0.0)	0 (0.0)	0.014
Comedications						
Antidepressants	117 555 (19.2)	58 832 (10.2)	0.257	4180 (4.8)	4108 (4.7)	0.004
Oral anticoagulants	7100 (1.2)	1007 (0.2)	0.122	19 (0.0)	24 (0.0)	−0.006
Antiplatelet agents	38 428 (6.3)	2908 (0.5)	0.323	75 (0.1)	53 (0.1)	0.011
NSAIDs	116 057 (18.9)	69 554 (12.0)	0.192	6579 (7.5)	6448 (7.4)	0.006
Antipsychotics	15 153 (2.5)	4941 (0.9)	0.127	199 (0.2)	175 (0.2)	0.006

Abbreviations: BMI, body mass index (calculated as weight in kilograms divided by height in meters squared); CAD, coronary artery disease; CHF, congestive heart failure; CKD, chronic kidney disease; NA, not applicable; NSAID, nonsteroidal anti-inflammatory drug; PS, propensity score; SMD, standardized mean difference; TBI, traumatic brain injury; TIA, transient ischemic attack.

^a^ Matching variables.

^b^ Not considered in PS matching model due to low prevalence (<0.1%).

Median (IQR) follow-up duration in the PS-matched cohort was 13.0 (8.7-18.5) years among individuals with influenza immunization at midlife and 11.5 (7.7-15.8) years among individuals without influenza immunization at midlife. During follow-up, 15.7% of individuals without influenza immunization at midlife were censored due to immunization for influenza (eTable 1 in Supplement 1). Influenza immunization at midlife, compared with a lack thereof, was not associated with the risk of PD (crude IRs per 1000 person-years, 0.16 vs 0.10; PS-matched HR, 0.96; 95% CI, 0.76-1.22) (Table 2; eTable 2 in Supplement 1). Cumulative incidence curves showed that approximately 10% of events occurred in the first 10 years of follow-up (eFigure 2 in Supplement 1).

**Table 2.** Propensity Score–Matched Hazard Ratios of Parkinson Disease Associated With Early Immunization for Influenza: Primary Analysis and Stratified by Demographic Characteristics

Characteristic	No.			IR per 1000 PY	PS-matched HR (95% CI)
	Individuals	Events	PY		
**Primary analysis**					
With influenza immunization at midlife	87 562	163	1 237 170	0.13	0.96 (0.76-1.22)
Without influenza immunization at midlife	87 562	122	1 078 194	0.11	1 [Reference]
**Age <45 y**					
With influenza immunization at midlife	46 347	57	639 469	0.09	1.00 (0.67-1.47)
Without influenza immunization at midlife	46 347	45	573 810	0.08	1 [Reference]
**Age ≥45 y**					
With influenza immunization at midlife	34 928	112	515 195	0.22	1.02 (0.75-1.38)
Without influenza immunization at midlife	34 928	72	430 364	0.17	1 [Reference]
**Female sex**					
With influenza immunization at midlife	50 078	81	726 198	0.11	0.83 (0.60-1.16)
Without influenza immunization at midlife	50 078	67	624 041	0.11	1 [Reference]
**Male sex**					
With influenza immunization at midlife	36 487	101	512 347	0.20	1.08 (0.79-1.48)
Without influenza immunization at midlife	36 487	69	450 777	0.15	1 [Reference]

Abbreviations: HR, hazard ratio; IR, incidence rate; PS, propensity score; PY, person-years.

In secondary analyses, stratification by age, sex, or vaccination prior to cohort entry did not modify the association between influenza immunization at midlife and risk of PD (Tables 2 and 3; eTable 2 in Supplement 1). However, seasonality was a potential effect size modifier (Table 3; eTable 2 in Supplement 1). Results varied over time, with the lowest point estimate approximately 8 years after vaccination (HR, 0.75; 95% CI, 0.52-1.08), but none of the differences were statistically significant. Results also varied by seasonality, with a lower point estimate for those vaccinated during influenza season (PS-matched HR, 0.62; 95% CI, 0.33-1.15) compared with those vaccinated outside of influenza season (matched HR, 1.07; 95% CI, 0.81-1.42).

**Table 3.** Propensity Score–Matched Hazard Ratios of Parkinson Disease Associated With Early Immunization for Influenza, Stratified by Vaccine-Related Effect Modifiers

Modifier	No.			IR per 1000 PY	PS-matched HR (95% CI)
	Individuals	Events	PY		
**During influenza season**					
With influenza immunization at midlife	12 313	21	173 162	0.12	0.62 (0.33-1.15)
Without influenza immunization at midlife	12 313	22	151 716	0.15	1 [Reference]
**Outside of influenza season**					
With influenza immunization at midlife	54 050	126	795 429	0.16	1.07 (0.81-1.42)
Without influenza immunization at midlife	54 050	83	681 650	0.12	1 [Reference]
**No vaccination before cohort entry**					
With influenza immunization at midlife	55 325	110	784 910	0.14	0.89 (0.67-1.18)
Without influenza immunization at midlife	55 325	91	684 755	0.13	1 [Reference]
**Vaccination before cohort entry**					
With influenza immunization at midlife	22 294	47	319 587	0.15	1.31 (0.81-2.11)
Without influenza immunization at midlife	22 294	25	275 249	0.09	1 [Reference]

Abbreviations: HR, hazard ratio; IR, incidence rate; PS, propensity score; PY, person-years.

In the time-response association analysis with time since influenza immunization at midlife serving as the underlying time axis, results varied over time but none of the differences were statistically significant (Figure). Specifically, there was a gradual decrease in the effect estimate of PD down to a trough HR of 0.75 (95% CI, 0.52-1.08) approximately 8 years after vaccination, albeit not reaching statistical significance with the 95% CI including the null value. Sensitivity analyses led to findings that were consistent with those of the primary analysis (HRs between 0.91 and 1.01; all statistically nonsignificant) (eTables 3-4 in Supplement 1). There was no clear dose-dependent association (eTable 5 in Supplement 1).

**Figure.**
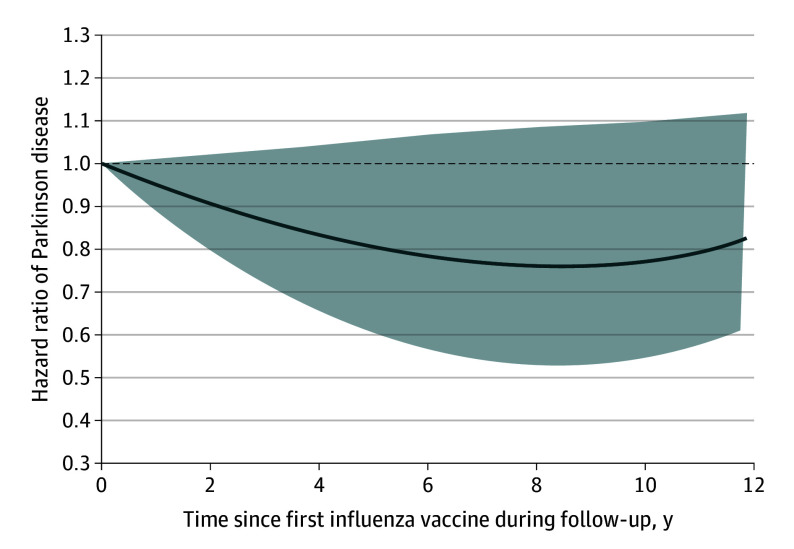
Time Since First Influenza Vaccine During Follow-Up Line indicates the hazard ratios, and shaded areas indicate the 95% CIs.

## Discussion

This large population-based cohort study of more than 1 million individuals showed that influenza immunization at midlife was not associated with the risk of incident PD overall. However, our findings suggest a potential time-response association with a gradual numerical decrease in the risk reaching a trough HR of 0.75 approximately 8 years after vaccination. We also observed a numerical decrease in the risk among individuals who were vaccinated during influenza season.

One of the earlier indications in favor of a potential association between the influenza virus and the risk of PD was the outbreak of encephalitic lethargica and postencephalitic Parkinsonism after the 1918 H1N1 influenza pandemic. Half a century later, in 1969, it was accidentally discovered that a patient with PD experienced improvement in their motor symptoms after receiving amantadine, a glutamate receptor antagonist also used in the treatment of influenza infection.^19^ In the next decades, amantadine became part of the therapeutic algorithms of PD.^20^

Similar to Alzheimer disease, another neurodegenerative condition of the central nervous system, influenza infection has been discussed as a potential etiology of PD.^5,21^ In addition to preclinical evidence, several observational studies have alluded to an association between influenza and an increased risk of this nigrostriatal disease.^8,9,10^ This association seems to be more prominent or even becoming apparent several years after the infection.^3,10^ For example, a case-control study based on Danish data spanning several decades assessed the association between “any influenza infection prior to PD” and the risk of PD; the study showed a numerical increase that did not reach statistical significance (odds ratio [OR], 1.28; 95% CI, 0.91-1.82).^10^ However, when stratifying based on time since infection, the Danish study showed that the increase in the risk of PD reached statistical significance 10 years after the infection (OR, 1.73; 95% CI, 1.11-2.71) and reached its peak magnitude 15 years after the infection (OR, 1.91; 95% CI, 1.14-3.19).

Our findings regarding the potential benefits of immunization for influenza at midlife for the risk of PD are in line with these observations. Similar to the study on the risk of PD with influenza infection,^10^ we also observed no association between immunization for influenza at midlife and the risk of PD in the overall population, but there was a potential time-dependent association. There was a numerical decrease in the risk of PD over time that reached a maximum of 25% approximately 8 years after the vaccination. This potential maximum decrease in the risk of PD associated with immunization at midlife would be smaller than the maximum increase in the risk of PD associated with influenza infection observed in the Danish data (91%).^10^ This finding is congruent with the relatively low effectiveness of influenza vaccines in preventing acute respiratory infections.^22^

Immunization for influenza at midlife was further associated with a numerical decrease in the risk of PD among individuals vaccinated during influenza season; this result was not observed among individuals vaccinated outside of influenza season. Given that the benefits of the vaccine for PD appear more plausible when mediated by the prevention of influenza than by alternative mechanisms, this finding might be in line with a causal association.

### Strengths and Limitations

Our study has several strengths. First, the large sample size (>1 million individuals) enabled the calculation of precise effect estimates of the potential association between immunization for influenza at midlife and the risk of PD in the primary and most secondary analyses. Second, the population-based character of the CPRD likely augmented the external validity of our findings. However, the requirement for at least 5 years of follow-up means that our findings may not necessarily be generalizable to individuals dying within the first few years after immunization for influenza at midlife. Third, the long follow-up spanning several decades allowed the consideration of the long pathogenic process of PD. It also allowed the application of a 2-year lag that minimized reverse causality and early detection bias.

Our study also has some potential limitations. First, due to the study’s observational design, residual confounding due to unmeasured confounders was possible. However, we went to great lengths to alleviate this bias by matching on demographic characteristics and on PS that included more potential confounders, thereby using a tight caliper. Second, our exposure definition was based on records of public health care practitioners, which could introduce some misclassification of influenza vaccine exposure and thus potentially dilute the true outcome. Nevertheless, we expect the majority of the UK population to receive vaccines from these practitioners given the nation’s universal health care program. Finally, misclassification of the outcome is also possible. However, the recording of PD in the CPRD has been shown to be accurate.^14^ Moreover, we conducted a sensitivity analysis using a stricter outcome definition that additionally required prescriptions for PD medications close to the diagnosis; this sensitivity analysis corroborated the results of the primary analysis.

## Conclusions

The results of this cohort study showed that influenza immunization at midlife was not associated with the risk of incident PD in the overall population. However, temporary decreases in PD risk several years after vaccination and decreases in PD risk among those vaccinated during influenza season appear possible, although the respective findings did not reach statistical significance and thus require further investigation and replication. Future studies should ideally involve even larger sample sizes that would facilitate analyses stratified by year to account for the outcome heterogeneity potentially resulting from the continual evolutionary changes in influenza viruses and the varying effectiveness of influenza vaccines.

## Notes

Supplement 1. **eFigure 1.** Flowchart Illustrating the Construction of the Study Cohort **eFigure 2.** Cumulative Incidence Curves for PD in the Matched Cohort **eTable 1.** Reasons for Censoring in the Two Exposure Groups **eTable 2.** Crude Hazard Ratios of PD Associated With Immunization for Influenza at Midlife (Primary and Secondary Analyses) **eTable 3.** PS Matched Hazard Ratios of PD Associated With Immunization for Influenza at Midlife (Sensitivity Analyses) **eTable 4.** Crude Hazard Ratios of PD Associated With Early Immunization for Influenza (Sensitivity Analyses) **eTable 5.** Crude and Adjusted Hazard Ratios of PD Associated With Immunization for Influenza at Midlife (Dose-Dependent Analyses)

Supplement 2.
Data Sharing Statement
