# Error in Key Points

**DOI:** 10.1001/jamanetworkopen.2025.56785

**Published:** 2026-01-08

In the Original Investigation titled “Influenza Immunization at Midlife and the Risk of Parkinson Disease,”^1^ published December 5, 2025, two words were missing in the Key Points Meaning section. The phrase starting “Findings of this study suggest that midlife….” should have been “ Findings of this study suggest that midlife influenza vaccination….” This article has been corrected.^1^
