# Supplementary material for: Influenza Immunization at Midlife and the Risk of Parkinson Disease
Source: JAMA Netw Open. 2025 Dec 5;8(12):e2547140. doi: 10.1001/jamanetworkopen.2025.47140 (PMC12681034; doi:10.1001/jamanetworkopen.2025.47140)
Supplement: Supplement 1. — eFigure 1. Flowchart Illustrating the Construction of the Study Cohort eFigure 2. Cumulative Incidence Curves for PD in the Matched Cohort eTable 1. Reasons for Censoring in the Two Exposure Groups eTable 2. Crude Hazard Ratios of PD Associated With Immunization for Influenza at Midlife (Primary and Secondary Analyses) eTable 3. PS Matched Hazard Ratios of PD Associated With Immunization for Influenza at Midlife (Sensitivity Analyses) eTable 4. Crude Hazard Ratios of PD Associated With Early Immunization for Influenza (Sensitivity Analyses) eTable 5. Crude and Adjusted Hazard Ratios of PD Associated With Immunization for Influenza at Midlife (Dose-Dependent Analyses) [file jamanetwopen-e2547140-s001.pdf]

Supplemental Online Content

Douros A, Cui Y, Dell'Aniello S, Suissa S, Brassard P. Influenza immunization at midlife and the risk of Parkinson disease. *JAMA Netw Open*. 2025;8(12):e2547140. doi:10.1001/jamanetworkopen.2025.47140

eFigure 1. Flowchart Illustrating the Construction of the Study Cohort

eFigure 2. Cumulative Incidence Curves for PD in the Matched Cohort

eTable 1. Reasons for Censoring in the Two Exposure Groups

eTable 2. Crude Hazard Ratios of PD Associated With Immunization for Influenza at Midlife (Primary and Secondary Analyses)

eTable 3. PS Matched Hazard Ratios of PD Associated With Immunization for Influenza at Midlife (Sensitivity Analyses)

eTable 4. Crude Hazard Ratios of PD Associated With Early Immunization for Influenza (Sensitivity Analyses)

eTable 5. Crude and Adjusted Hazard Ratios of PD Associated With Immunization for Influenza at Midlife (Dose-Dependent Analyses)

This supplemental material has been provided by the authors to give readers additional information about their work.

© 2025 Douros A et al. *JAMA Network Open*

eFigure 1. Flowchart illustrating the construction of the study cohort

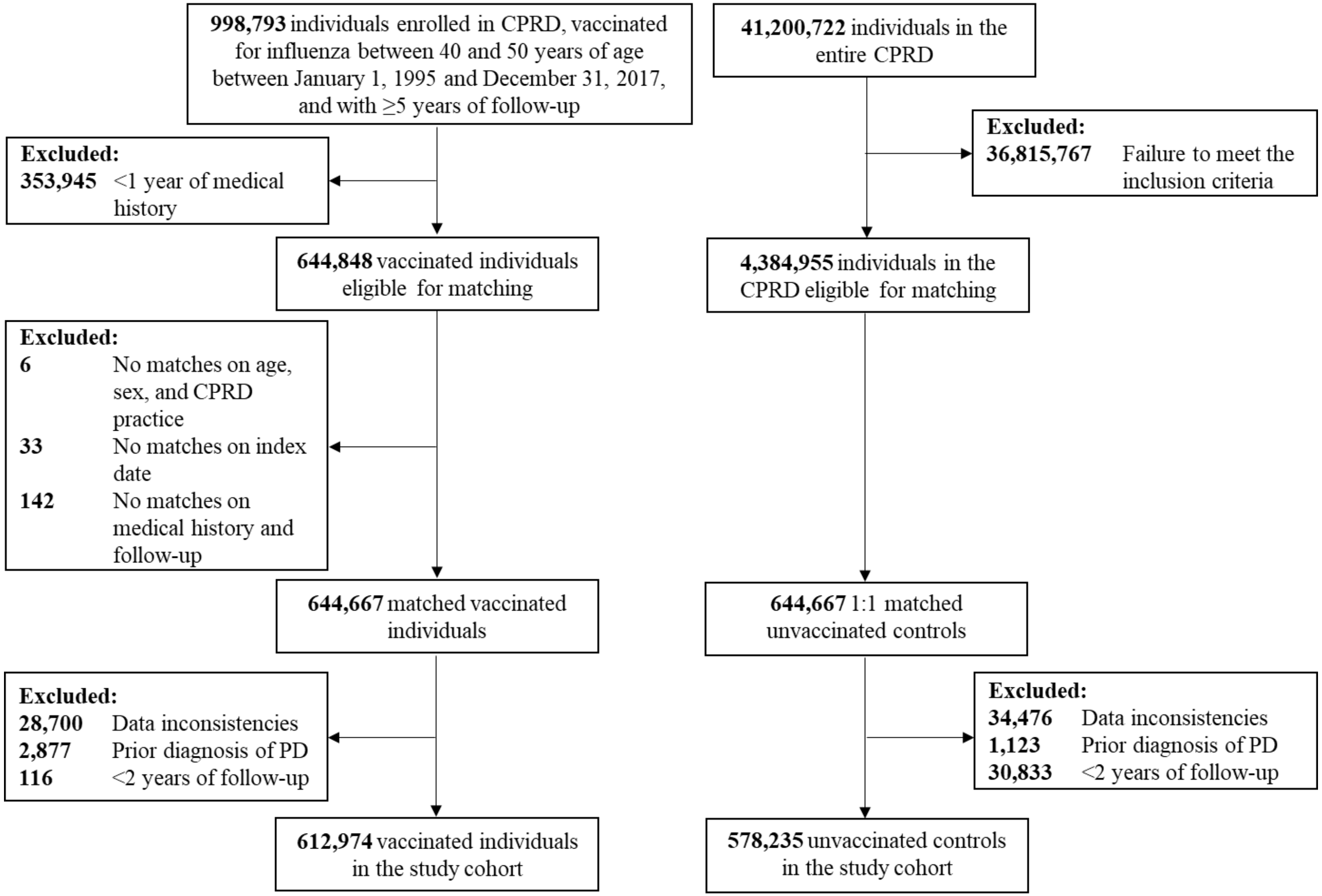

© 2025 Douros A et al. *JAMA Network Open*

Abbreviations: CPRD, Clinical Practice research Datalink.; PD, Parkinson’s disease

© 2025 Douros A et al. *JAMA Network Open*

eFigure 2. Cumulative incidence curves for PD in the matched cohort

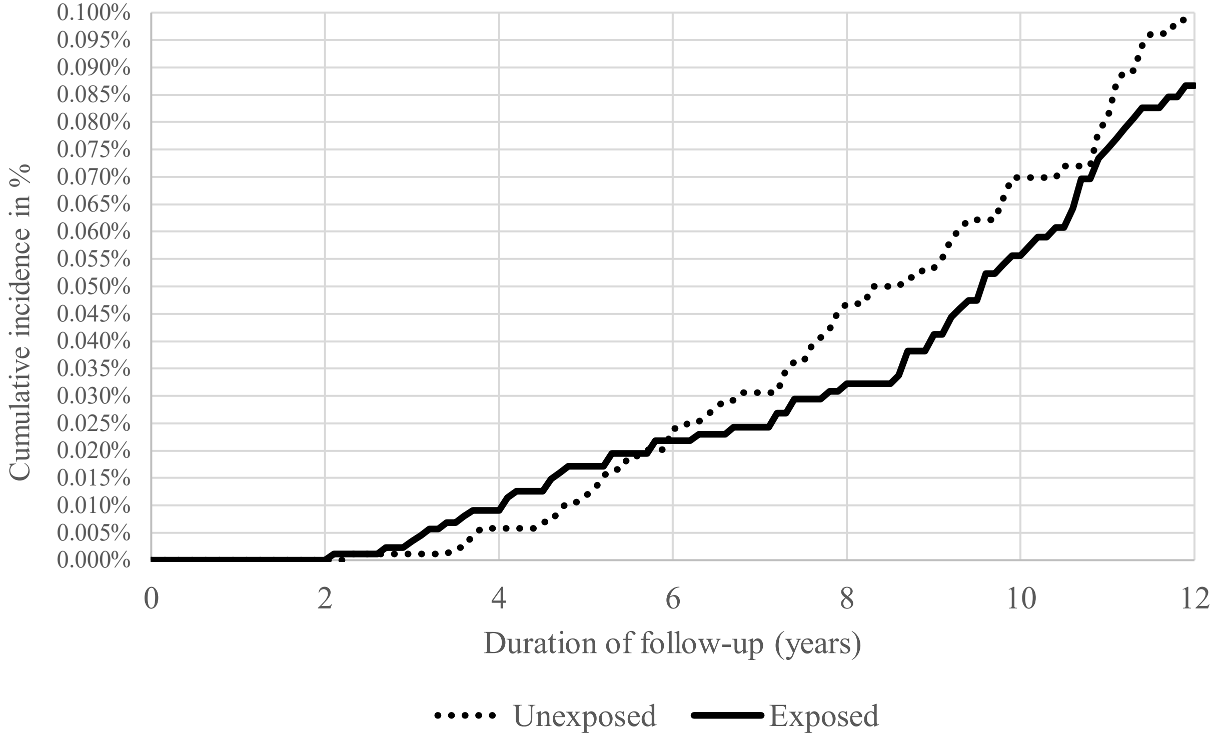

Exposed	87,562	87,562	87,548	84,546	71,366	59,450	48,937
Unexposed	87,562	87,562	85,306	79,762	64,685	51,409	40,338

© 2025 Douros A et al. *JAMA Network Open*

Abbreviations: PD, Parkinson’s disease.

© 2025 Douros A et al. *JAMA Network Open*

eTable 1. Reasons for censoring in the two exposure groups

	N Individuals	Death n (%)	Treatment switch n (%)	Administrative censoring n (%)	Outcome n (%)
Exposed	87,562	4,294 (4.90)	NA	83,105 (94.91)	163 (0.19)
Unexposed	87,562	2,093 (2.39)	13,712 (15.66)	71,635 (81.81)	122 (0.14)

Abbreviations: NA, not applicable.

© 2025 Douros A et al. *JAMA Network Open*

eTable 2. Crude hazard ratios of PD associated with immunization for influenza at midlife (primary and secondary analyses)

	N Individuals	N Events	N PY	IR*	Crude HR (95% CI)
**Primary analysis**					
Exposed	612,974	1,313	8,319,495	0.16	1.34 (1.22-1.48)
Unexposed	578,235	661	6,833,106	0.10	1.00 (reference)
**Age <45 years**					
Exposed	336,229	409	4,452,089	0.09	1.21 (1.03-1.42)
Unexposed	316,136	249	3,754,455	0.07	1.00 (reference)
**Age ≥45 years**					
Exposed	276,745	904	3,867,406	0.23	1.36 (1.21-1.54)
Unexposed	262,099	412	3,078,651	0.13	1.00 (reference)
**Female sex**					
Exposed	348,608	545	4,745,678	0.11	1.27 (1.09-1.47)
Unexposed	325,312	285	3,829,083	0.07	1.00 (reference)
**Male sex**					
Exposed	264,366	768	3,573,817	0.21	1.42 (1.25-1.62)
Unexposed	252,923	376	3,004,023	0.13	1.00 (reference)
**During influenza season**					
Exposed	90,831	177	1,221,844	0.14	1.38 (1.06-1.79)
Unexposed	85,655	84	1,011,585	0.08	1.00 (reference)
**Out of influenza season**					
Exposed	367,728	945	5,204,066	0.18	1.37 (1.22-1.54)
Unexposed	346,537	452	4,192,737	0.11	1.00 (reference)
**No vaccine before cohort entry**					
Exposed	413,785	887	5,605,275	0.16	1.23 (1.10-1.38)
Unexposed	401,832	518	4,745,022	0.11	1.00 (reference)
**Vaccine before cohort entry**					
Exposed	199,189	426	2,714,220	0.16	1.76 (1.45-2.13)
Unexposed	176,403	143	2,088,084	0.07	1.00 (reference)

Abbreviations: PD, Parkinson's disease; HR, hazard ratio; PY, person-years; IR, incidence rate; CI, confidence interval; IPCW, inverse probability of censoring weighting.

* Calculated per 1,000 PY.

© 2025 Douros A et al. *JAMA Network Open*

eTable 3. PS matched hazard ratios of PD associated with immunization for influenza at midlife (sensitivity analyses)

	N Individuals	N Events	N PY	IR*	PS matched HR (95% CI)
**3-year lag period**					
Exposed	85,954	159	1,213,042	0.13	1.09 (0.85-1.39)
Unexposed	85,954	121	1,071,261	0.11	1.00 (reference)
**5-year lag period**					
Exposed	84,806	142	1,198,300	0.12	1.06 (0.82-1.36)
Unexposed	84,806	115	1,080,451	0.11	1.00 (reference)
**10-year lag period**					
Exposed	44,918	87	764,638	0.11	1.16 (0.84-1.59)
Unexposed	44,918	67	716,917	0.09	1.00 (reference)
**Stricter outcome definition**					
Exposed	87,569	143	1,237,417	0.12	1.16 (0.90-1.51)
Unexposed	87,569	101	1,078,374	0.09	1.00 (reference)
**No IPCW**					
Exposed	87,481	122	1,107,842	0.11	0.92 (0.72-1.19)
Unexposed	87,481	122	1,076,354	0.11	1.00 (reference)
**Excluding unexposed with prior vaccine**					
Exposed	83,141	157	1,183,543	0.13	1.04 (0.82-1.33)
Unexposed	83,141	122	1,035,177	0.12	1.00 (reference)

Abbreviations: PD, Parkinson's disease; PS, propensity score; HR, hazard ratio; PY, person-years; IR, incidence rate; CI, confidence interval; IPCW, inverse probability of censoring weighting.

* Calculated per 1,000 PY.

© 2025 Douros A et al. *JAMA Network Open*

eTable 4. Crude hazard ratios of PD associated with early immunization for influenza (sensitivity analyses)

	N Individuals	N Events	N PY	IR*	Crude HR (95% CI)
**3-year lag period**					
Exposed	612,921	1,281	8,319,360	0.15	1.35 (1.22-1.49)
Unexposed	568,109	636	6,807,951	0.09	1.00 (reference)
**5-year lag period**					
Exposed	612,479	1,200	8,317,326	0.14	1.34 (1.21-1.48)
Unexposed	550,600	587	6,738,298	0.09	1.00 (reference)
**10-year lag period**					
Exposed	401,044	937	6,693,674	0.14	1.40 (1.24-1.58)
Unexposed	320,430	399	4,993,501	0.08	1.00 (reference)
**Stricter outcome definition**					
Exposed	612,997	1,093	8,321,113	0.13	1.24 (1.12-1.38)
Unexposed	578,251	591	6,833,615	0.09	1.00 (reference)
**No IPCW**					
Exposed	583,705	982	7,144,274	0.14	1.33 (1.20-1.47)
Unexposed	578,235	661	6,833,106	0.10	1.00 (reference)
**Excluding unexposed with past vaccination**					
Exposed	584,413	1,277	7,985,028	0.16	1.33 (1.21-1.47)
Unexposed	548,084	651	6,547,901	0.10	1.00 (reference)

Abbreviations: PD, Parkinson's disease; HR, hazard ratio; PY, person-years; IR, incidence rate; CI, confidence interval; IPCW, inverse probability of censoring weighting.

* Calculated per 1,000 PY.

© 2025 Douros A et al. *JAMA Network Open*

eTable 5. Crude and adjusted hazard ratios of PD associated with immunization for influenza at midlife (dose-dependent analyses)

	N Episodes	N Events	N PY	IR*	Crude HR (95% CI)	Adjusted HR (95% CI)
**Time-dependent Cox model****						
≥8 vaccinations	279,946	811	1,768,094	0.46	1.59 (1.41-1.79)	1.38 (1.20-1.59)
2-7 vaccinations	638,103	647	3,445,417	0.19	1.31 (1.16-1.47)	1.21 (1.07-1.38)
1 vaccination	864,928	321	4,429,542	0.07	1.02 (0.88-1.17)	0.99 (0.85-1.14)
0 vaccinations	609,005	669	6,935,888	0.10	1.00 (reference)	1.00 (reference)
**Marginal structural Cox model*****						
≥8 vaccinations	279,946	811	1,768,094	0.46	1.59 (1.41-1.79)	1.20 (1.03-1.40)
2-7 vaccinations	638,103	647	3,445,417	0.19	1.31 (1.16-1.47)	1.09 (0.95-1.25)
1 vaccination	864,928	321	4,429,542	0.07	1.02 (0.88-1.17)	1.01 (0.86-1.17)
0 vaccinations	609,005	669	6,935,888	0.10	1.00 (reference)	1.00 (reference)

Abbreviations: PD, Parkinson's disease; HR, hazard ratio; PY, person-years; IR, incidence rate; CI, confidence interval.

* Calculated per 1,000 PY.
** Adjusted for all baseline covariates.
*** Adjusted for all baseline covariates and additionally for body mass index and comedications as time-dependent covariates updated every year.

© 2025 Douros A et al. *JAMA Network Open*
