# Supplementary material for: Influenza Immunization at Midlife and the Risk of Parkinson Disease
Source: JAMA Netw Open. 2025 Dec 5;8(12):e2547140. doi: 10.1001/jamanetworkopen.2025.47140 (PMC12681034; doi:10.1001/jamanetworkopen.2025.47140)
Supplement: Supplement 2. — Data Sharing Statement [file jamanetwopen-e2547140-s002.pdf]

Data Sharing Statement

Douros. Influenza Immunization at Midlife and the Risk of Parkinson Disease. *JAMA Netw Open*. Published December 05, 2025. doi:10.1001/jamanetworkopen.2025.47140

Data
Data available: No

Additional Information
Explanation for why data not available: This study is based in part on data from the CPRD obtained under license from the UK Medicines and Healthcare products Regulatory Agency. The data are provided by patients and collected by the UK National Health Service as part of their care and support. The interpretation and conclusions contained in this study are those of the authors alone. Because electronic health records are classified as "sensitive data" by the UK Data Protection Act, information governance restrictions (to protect patient confidentiality) prevent data sharing via public deposition. Data are available with approval through the individual constituent entities controlling access to the data. Specifically, the primary care data can be requested via application to the Clinical Practice Research Datalink (https://www.cprd.com).
